# Miscellaneous

**Published:** 1876-01

**Authors:** 


### Miscellaneous.

### THE CARE OF THE EYES IN CHILDREN.

The *Sanitarian* quotes the following extract from a late work of Dr. C. R. Agnew:

Any malady which deprives a growing child of its natural, playful exercises, and shuts it up in the poisonous atmosphere of an imperfectly lighted and ventilated, or over-heated domicile, is eminently worthy of our earnest and thoughtful attention.

The more intractable forms of these diseases—phlyctenular, or so called strumous ophthalmia—occur in children who may have inherited feeble constitutions. In many instances, however, they occur in those who have been born without any special constitutional defect, but who have had their digestive and other blood making organs injured by a promiscuous dietary, and too little out-of-door life. The practice, common in every walk of life, of giving the diet of the family table to a young child so soon as it can stretch out its hands to gratify its natural gluttonous propensities, is here seen to produce one of its many evil results. Such remarks may seem to some to be trite, but they will not cease to be pertinent so long as the evil at which they are aimed continues to be so wide-spread. If children, when weaned, were put first upon a milk, and then a milk and farinaceous diet, and not allowed to have the full, promiscuous dietary of adult life, we would see a vast improvement in public health and a lower death rate. We would, moreover, see less of those forms of self-indulgence which spring from the uncontrolled selfishness and gluttony that begin at the nursery table.

We are prepared to urge, then, in every case of phlyctenular or eruptive inflammation of the conjunctiva, that the diet and regimen be placed upon a hygienic basis. We should also prescribe woolen under clothing, with a change of the same for the night, that the temperature of the domicile need not be kept above 65 degrees. We should improve the action of the skin by alkaline or saline baths and daily friction. We should insist upon daily out-of-door life, and keep the child, when domiciled, in a well-lighted room, warmed, or at least ventilated, if possible, by an open fire in winter, and its eyes free from eyeshades, bandages, poultices and sugar of lead washes. When I say well-lighted rooms, I do not mean those middle or inside rooms in which so many people spend so much time, or in which they incarcerate their children, but outside rooms, that are penetrated and purified by direct sunlight. A child should never be allowed, sick or well, to play long or sleep in a room that is not above the ground level and daily pervaded by direct sunlight. People who value the lives of their children should select their homes with these things in view.

### ON MEAT AND MILK.

The *Doctor* says:—M. Leven, who has investigated, with much patience, various problems involved in the digestion of food, has drawn from his experiments some practical conclusions which deserve the attention of all practitioners. He says that, in order to ascertain the digestibility of food, we must find out not only what is its aptitude to undergo the action of the digestive juices, but also what effect the food produces on the mucous membrane.

We must know in what part of the digestive tube the food is elaborated, whether in the stomach or in the intestine. Thus, meat stays a long time in the stomach, and milk a very short time. So that, if we have to treat an ulcer in the stomach, we can easily understand how important it is not to give meat, which excites the contraction of the muscular fibers of the stomach, and the circulation of the mucous membrane, in order to avoid returns of hæmatemesis.

Milk, on the contrary, will be preferred; it is an inert substance, which leaves the stomaeh to repose, and glides softly through the pylorus without any effort of the organ. It has been said, in such cases, that milk is more digestible than meat; but are we authorized to make this statement? No one has measured the time necessary for milk to pass into the circulation. How many hours does it take to emulsionize fat, and to render casein absorbable? Does milk pass into the blood quicker than meat? That is probable; but we cannot, and ought not, to establish any comparison between the digestibility of these two substances. It does not take place on the same spot, and we have to do with perfectly different substances, between which we ought to make no comparison. Milk, we have said, leaves the stomach at rest; meat, on the contrary, keeps it in a necessary condition of excitation, which is useful with regard to digestion in general. If we prolong feeding on milk diet, we bring on a condition of weakness and apathy of the stomach, and it will easily be deranged by the slightest change of diet.

Meat, on the contrary, keeps up the activity of the stomach, comforts it, and in a general way facilitates the digestion. It is thus that, without considering substances with respect to their digestibility, it is important to know in what conditions their digestion takes place, in order that we may lay down the indications for hygiene and clinical practice.

### EVILS FROM THE USE OF WHEATEN FLOUR.

In the *Transactions* of the New Hampshire Medical Society, Dr. Ephraim Cutter, of Cambridge, Mass., brings a heavy indictment against flour as a food. His article aims to answer in the affirmative all the following questions:—

1. May it not be possible that the use of flour is a cause of the prevalence of diseases of the nervous system?

2. May it not be possible that the use of flour is a cause of the present lamentable and astounding prevalence of late erupting and decayed teeth?

3. May it not be possible that the use of flour is one cause of the present prevalence of weak and diseased eyes?

4. May it not be possible that the use of flour is one cause of the prevalence of baldness and premature gray hair?

5. May it not be possible that the so-called change in type of disease may, in some measure, be due to the use of flour, so universal for the past forty years.

6. May it not be possible that the use of flour is one cause of the prevalence of some of our chronic diseases, as catarrh and consumption?

7. May it not be possible that the use of flour is one cause of the numerical decline in our native population in New England?

He concludes, that the universal and exclusive use of flour as found at the present time among the nations of Christendom (as Liebig suggests), results in disaster to the human race in these particulars.

### PEROXIDE OF HYDROGEN AS A DEODORIZER.

In a recent lecture on deodorizers and disinfectants, by Dr. John Day, the speaker said that the great value of peroxide of hydrogen as a deodorizer was only just beginning to be understood. Professor Roscoe had stated, as recently as 1871, that hydrogen dioxide, or peroxide, "does not occur in nature." Dr. Day claimed, however, to have discovered its presence, spontaneously generated, in a vast number of substances which are in almost daily use, such as all fats and fatty or expressed oils; nearly all perfumes; most, if not all, essential oils; kerosene, gasoline and benzine; and deal and pine woods. In many respects, gasoline is the best disinfectant with which he is acquainted. In addition to being highly volatile, it possessed the property of either generating peroxide of hydrogen, or of originally forming it, and storing it up until brought into contact with any of those oxidizable substances for which it has an affinity. One of these two actions, he could not say which, took place long after all presence of the gasoline itself passed away. When unglazed paper, or any porous substance, is brushed over with gasoline, it will at once give the reaction of peroxide of hydrogen and continue to do so for a year or more. It is thus persistent in its action, which gives it an immense value over other disinfectants. He recommends that books, newspapers, etc., which have been used by fever patients or kept in their apartments, be disinfected by brushing them over with gasoline. The most delicate wall paper may be brushed over with it without injury, as may articles of wearing apparel. The hands may be disinfected by brushing them over with gasoline and allowing them to dry in the air. There is one drawback to its use—viz., its inflammability. Nevertheless gasoline appears to be worthy of more attention as a disinfectant than it has hitherto received.

### THE DETECTION OF ARSENIC.

Often when chemical analysis has succeeded in detecting the existence of arsenic in the tissues, it has failed to determine the exact quantity. M. Armand Gautier, director of the chemical laboratory, at the School of Medicine, Paris, has undertaken the task and he believes he has succeeded in meeting the desideratum. His method consists in totally destroying the animal tissues, so as to isolate the arsenic. This is done with pure nitric acid, followed by the addition of sulphuric acid, and then the destruction is completed by again having recourse to nitric acid. To the liquid thus obtained, a few drops of the bisulphite of soda are added, and the sulphuret of arsenic is then precipitated in the ordinary way by sulphuretted hydrogen. M. Gautier has, in this way, completely isolated the entire quantity of arsenic he had himself introduced into the bodies of different animals. In the course of his experiments, M. Gautier made one very important discovery, viz., that the arsenic is first localized in the nervous system and then passes on to the liver and the muscles.

### SPARE THE IVY.

The *Sanitarian* says, the English ivy, growing over the walls of a building, instead of promoting dampness, as many persons would suppose, is said to be a remedy for it; and it is mentioned as a fact, that in a room where damp had prevailed for a length of time, the affected parts inside had become dry when ivy had grown up to cover the opposite exterior side. The close, overhanging pendant leaves prevent the rain or moisture from penetrating the wall. Beauty and utility, in this case go hand in hand.

### DISINFECTANTS AND DISINFECTION.

An interesting paper by E. Waller, Ph. D., in the *American Chemist* for July, sums up admirably what is at present definitely known on this important subject. The following are the conclusions drawn by the writer:

1. For prompt disinfection which is to be only temporary, strong oxydizing agents, as chlorine, permanganate of potassium, nitric acid, etc., should be used. Of these the cheapest and most available is the chloride of lime.

2. Carbolic acid may be diluted to a point at which it ceases to act as a disinfectant, though it may still act as an antiseptic. To disinfect, the carbolic acid should probably constitute about one per cent of the mixture.

3. The action of metallic solutions in disinfecting is comparatively slow. Ferric nitrate especially, and iron and zinc salts generally, are the best of this class of disinfectants.

4. A mixture of iron and zinc salts seems to be superior to either used alone.

5. The preparations known as carbolates of lime usually contain but little carbolic acid, and are of comparatively little efficacy. The lime absorbs carbonic acid from the air, and the efficiency of these preparations as disinfectants is apparently diminished in proportion as the lime assumes the form of carbonate.

6. The powders (carbolated) containing no lime have still less effect on putrid matter than those in which lime is an essential constituent.

### WHO SHALL CONFER DEGREES?

The New York *Medical Record* of 28th ult. has an editorial on professional education, the pith of which is contained in the following paragraph: "We believe that the foundation of all reform in medical education rests mainly upon the enforcement of the two conditions, the necessity of which we have so frequently pointed out,—viz: a proper preliminary education, and an independent examining board. These two points are the *alpha* and *omega* of the requirements of the prospective practitioner, while the office of the medical college is merely to fill up the gap between the two."

While admitting that the colleges have some selfish interest in graduating their students, we do not see how, with human nature in its present state of improvement, the matter can be much bettered by the adoption of the autocratic system of foreign governments. If the trustees of colleges cannot be trusted with the selection of professors competent to examine candidates for a degree, who shall have the authority to appoint examiners? and *who shall examine the examiners*?

It is a well-known fact that offices of State appointment are more liable to be filled with incapables than with properly qualified persons; and common experience is that examinations conducted by State Boards are generally ridiculous farces, and that the members are usually no more unselfish than the parties complained of by the *Record.* Anything that the law can do towards elevating the standard in any of the learned professions can be better done through the colleges than in any other way. Let the duration of the course of tuition be legthened by legal enactment if the people are dissatisfied with the present requirements. It seems to us that that is the only way to effectually secure a higher standard of education.—*Quericus, in Pharmacal Gazette.*

### THE DIGESTIVE MOVEMENT IN PLANTS.

The following account of this movement is that given by Dr. Darwin in his late work:

It opens up to us the secret of many of the so called animal motions. The interior of each cell is filled with, or rather is composed of, a mass of protoplasm. This at rest and in health is more or less uniform in character; but being irritated or excited, it becomes cloudy from the formation of granules, and these granules, in accordance with their ordinary laws, tend to aggregate into a spherical mass, leaving a clear ring of moving protoplasm around. This ring also disappears, and now there is but one central protoplasmic mass, undergoing all manner of changes of shape, such as we are wont to see in free-moving leucocytes. Cell after cell is thus affected in a moment's time, until, under the influence of the combined movements, the whole tentacle obeys the impulse, and closes in upon its prey. This continues whilst the gland cells are in an actively oxygenated state, but by-and-by ceases, the tentacles re-expand, the cellular masses of protoplasm are re-dissolved, and the cells fill with a homogeneous liquid.

### THE HEALING ELEMENT IN ARNICA.

The ingredient in arnica long suppossed most important was *arnicine*, an amorphous bitter substance, almost insoluble in water, but freely soluble in alcohol and ether; or else the ethereal oil, which is also insoluble in water. For a variety of reasons, it is now probable that neither arnicine nor the oil, but *trimethylamine*, an organic alkali, is the really useful constituent of arnica.

Trimethylamine, $C_3 H_9 N$, is a clear, colorless fluid, very volatile, and freely soluble in water, alcohol, and ether.

For external bruises and cuts arnica is, undoubtedly, very useful; and the mischances that have attended its use have probably resulted from the fact that the tincture, containing arnicine and the volatile oil, has been employed. The infusion or decoction alone should be used, and it would be better to give up employing all liniments and lotions in which the tincture is present.

